# Urinary cancer detection by the target urine volatile organic compounds biosensor platform

**DOI:** 10.1038/s41598-024-54138-1

**Published:** 2024-02-12

**Authors:** Vichayut Suthat Na Ayutaya, Chaianan Tantisatirapoon, Sekdusit Aekgawong, Watcharapong Anakkamatee, Taechasit Danjittrong, Chatchai Kreepala

**Affiliations:** 1 School of Internal Medicine, Institute of Medicine, Suranaree University of Technology, Nakhon Ratchasima, Thailand; 2 School of Surgery, Institute of Medicine, Suranaree University of Technology, Nakhon Ratchasima, Thailand; 3 Department of Mathematics, Faculty of Science, Naresuan University, Phitsanulok, Thailand; 4 Department of Anesthesiology, Chulabhorn Hospital, Bangkok, Thailand

**Keywords:** VOCs, Biosensor, Genitourinary cancer, Biotechnology, Cancer, Biomarkers, Health care, Nephrology, Oncology, Urology

## Abstract

Volatile organic compounds (VOCs) have grown due to their crucial role in transitioning from invasive to noninvasive cancer diagnostic methods. This study aimed to assess the feasibility of the metal oxide biosensor platform using urine VOCs for detecting genitourinary cancers. Five different commercially available semiconductor sensors were chosen to detect specific VOCs (methane, iso-butane, hydrogen, ethanol, hydrogen sulfide, ammonia, toluene, butane, propane, trimethylamine, and methyl-mercaptan). Changes in electrical resistance due to temperature variations from the voltage heater were examined to characterize VOC metabolism. Logistic regression and ROC analysis were employed to evaluate potential urine VOCs for genitourinary cancer determination. This study involved 64 participants which were categorized into a cancer and a non-cancer group. The genitourinary cancer (confirmed by tissue pathology) comprised 32 patients, including renal cell carcinoma (3.1%), transitional cell carcinoma (46.9%), and prostate cancer (50%). The non-cancer comprised 32 patients, with 9 healthy subjects and 23 individuals with other genitourinary diseases. Results indicated that VOC sensors for methane, iso-butane, hydrogen, and ethanol, at a voltage heater of 2000 mV, demonstrated a significant predictive capability for genitourinary cancer with *P* = 0.013. The ROC of these biomarkers also indicated statistical significance in predicting the occurrence of the disease (*P* < 0.05). This report suggested that methane, iso-butane, hydrogen, and ethanol VOCs exhibited potential for diagnosing genitourinary cancer. Developing gas metal oxide sensors tailored to these compounds, and monitoring changes in electrical resistance, could serve as an innovative tool for identifying this specific type of cancer.

## Introduction

Genitourinary malignancies are widespread and include a wide range of diseases, from mild benign tumors to deadly aggressive neoplasms. The most common urinary tract cancer is bladder cancer. The clinical issues in bladder cancer are early detection and proper follow-ups because a recurrence is common, and a delayed diagnosis is linked to a poor prognosis. In addition to playing a crucial role in bladder cancer patient management, the primary care physician may also be in charge of maintaining proper continuous surveillance^1^. There are several new techniques for finding urine biomarkers. They identify different proteins in urine that have been exposed to neoplasia and circumvent the limitations of the currently used treatments. Several biomarkers were developed for assays, including bladder tumor antigen and nuclear matrix protein-22, etc. However, they are not used widely due to their limited utility and the specificity has been proven to be low.

Volatile organic compounds (VOCs) have been increasing in significance in recent years due to their pivotal role in the transition from invasive to noninvasive cancer diagnosis techniques. With numerous efforts, there have been no effective non-invasive methods to diagnose cancers in their early stages. Therefore, new non-invasive techniques must be developed^2^.

The VOCs are released by the body and may serve as "Odor-fingerprints" of one's metabolic state. When exposed to room temperature, the organic chemical molecules known as VOCs readily evaporate^3^. VOCs are found in breath, blood, skin, and urine and may now be quickly and accurately analyzed as a result of recent advancements in analytical techniques. Infectious diseases, metabolic diseases, genetic disorders, and other diseases can all be diagnosed using disease-specific VOCs as olfactory biomarkers. Finding the pathophysiological mechanisms that cause the generation of disease-specific VOCs could offer fresh perspectives on the therapeutic approaches for treating various diseases^4^. Gas chromatography (GC) and gas chromatography and mass spectrometry (GC–MS) have both been employed to identify VOCs during the second half of the twentieth century. These cutting-edge analytical methods have made it possible for studies to concentrate on identifying VOCs that indicate particular smells from patients with each distinct illness. It could be possible to diagnose diseases using the detected VOC molecules as biomarkers^2,4^.

The urine volatile organic chemicals in urologic malignancies such as bladder, prostate, and renal cancer were investigated in several earlier research^5–8^. Furthermore, pattern recognition methods had been more frequently used in research of VOCs in bladder cancer to date^2,6^. It was discovered that particular types of VOCs, including phenol, decanal, nonanal, aniline, styrene, and dimethylsilanediol, have been emitted by the renal cell carcinoma by employing a chemical analytical approach, such as solid-phase microextraction (SPME) and GC–MS. Alkanes, aldehydes, and aromatic chemicals including 1,2,4-trimethylbenzene, 1-methylnaphthalene, and 2-methylnaphthalene have been associated with stomach cancer^9^. Additionally, decanoic acid and pentadecane-2-one are linked to prostate cancer^10^.

Nowadays, the development of semiconductor metal oxide sensors allows the detection of VOCs from breath and urine by relying on the change of voltage and electrical resistance. The studies use electric current to evaporate VOCs and focus on the changes of evaporation at different temperatures^11–14^. The results from the previous studies had shown that the changes in the voltage and electrical current resistance dynamics in the semiconductor metal oxide sensors indicate specific patterns that are specific to different types of VOCs. Therefore, this could transit the odor fingerprint in the biogas expression platform to the electrical expression platform due to the possibility of specific-disease VOC identification and the cost-effectiveness of the electrical expression platform when compared to SPME and GC–MS. The previous validation reports, comparing the accuracy of measurements by metal oxide and gas chromatography, were studied. The data indicated that the measurement results of both the main method and alternative methods, although differing in specimen preparation, could be applied interchangeably for detecting and measuring VOCs, maintaining sensitivity and specificity^15,16^.

The authors determined to use the knowledge of the change of voltages in the common VOCs to study the voltage dynamics that are specific in genitourinary cancer for screening the common cancers such as bladder cancer, prostate cancer, and renal cell carcinoma by using the available commercial semiconductor sensors. Regarding the fact that there were only a few studies on VOCs and genitourinary cancer, the authors therefore compared the properties of the VOCs of the urine from other renal diseases and also the VOCs from the urine in the normal population to use this collective data for the development of the detection of genitourinary cancer by using semiconductor sensors.

## Materials and methods

### Data collection

This was a descriptive cohort study conducted among 64 subjects attending outpatient clinic at Suranaree University of Technology Hospital during August 2021–July 2023. All the subjects were advised and voluntarily signed a consent form before participating in our study. The patients with renal diseases and the normal subjects were prohibited from having strong odor food (such as tea, coffee, onion, garlic, shrimp paste, acacia, fermented fish, pakria, and celery, etc.) for at least 3 h before the examination of urine VOCs and were prohibited alcohol intakes for more than 24 h before the urine VOCs examination. The urine samples were collected for 20 mL in universal bottles and samples were classified as genitourinary (kidney/bladder/prostate) cancer or non-cancer or normal control after pathological examination of the biopsy specimens. The urine samples were kept at room temperature (the temperature-controlled examination room) and were taken to be analyzed with the semiconductor sensors within 30 min after the samples were collected.

From the previous studies, the accuracies of using VOCs in the urine of patients with kidney cancer, bladder cancer, and prostate cancer, when compared to the normal population^8,9,17^, to differentiate the cancer were different among the cancers. The mean accuracies of using VOCs in renal cancer, bladder cancer, and prostate cancer compared to the normal population were 70.2%, 93.3%, and 65% respectively. The authors used the mean accuracies of VOCs from the previous studies as references to calculate the sample sizes of this study. By using the estimate multiple mean^18,19^ by determining the significance level (alpha or α) at 5% and 80% chance of detecting an effect size of 20% or greater respectively, the sample size in each population group was 20 samples for cancer and cancer-free subjects.

### Gas sensors

The authors selected five different commercially available semiconductor metal oxide sensors to detect all the targeted VOCs, which were methane, iso-butane, hydrogen, ethanol, hydrogen sulfide, ammonia, toluene, butane, propane, trimethylamine, methyl-mercaptan (manufactured by Figaro company). These were the types of VOCs that had been studied and were shown to be related to the malignant according to the literature. The detailed products and basic semiconductor circuits were available in the Figaro Product Information Manual (https://www.figarosensor.com/product/sensor/).

The method of analyzing the VOCs was by analyzing the evaporated VOCs from the urine in the container when they evaporated into the sealed chamber. The VOCs in the chamber were then analyzed at the same time by the five different gas semiconductor sensors which were located on the top of the chamber.

### Gas sensor analysis

All gas sensors that were placed in a sensor chamber were the n-type sensors. The VOCs that occurred from the metabolism of the cells were the reduction gas, therefore when the targeted VOCs evaporated into the conducting chamber, the targeted VOCs reacted with the oxygen ions in the gas sensors and released the free electrons. The phenomenon of free electron release causes a decrease in the electrical resistance within the conducting chambers, making the increase in a conducting property. Moreover, the different temperatures inside the conducting chamber affected the decomposition of the metal oxide into the free electrons in a different way. The author invented the conducting chamber by using the commercially available metal dioxide sensors manufactured by Figaro company. The operating voltage of the heater could produce heat equal to 5000 ± 200 mV DC, which was recommended by the manufacturer, to the metal dioxide sensors. Moreover, the electric voltage could produce five different heat energy which were 2000 mV, 2500 mV, 3500 mV, 4500 mV and 5000 mV in every 80 s. After that, the electrical resistance would then be measured in each sensor (S1, S2, S3……, S5).

**Figure 1:**
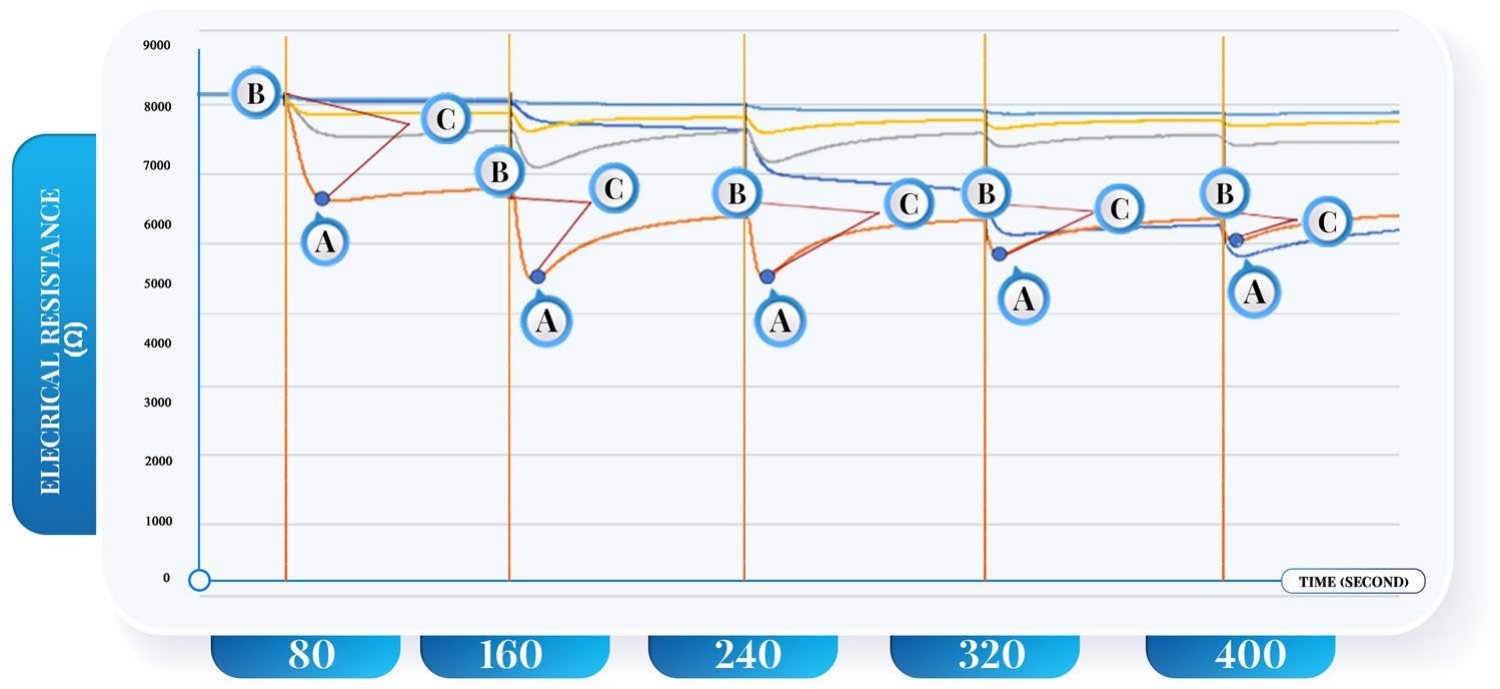
**the Gas Sensor Analysis from the change of the electrical resistance in each sensor:** Label sensor 2 (orange line) A = min or the lowest point of the electrical resistance of the voltage heater in each time which had five values min1- min5, C = the slope of the graph from plotting the time against the decrease of the electrical resistance. The C (the highest to the lowest) in each time had 5 values which were sl1-sl5. The delta resistance from point B to point A was equal to gap which had 5 values: gap1-gap5, time from B to A was equal to t which had 5 valves (t1-t5). **Abbreviation:** Ω = ohm; *the unit of electrical resistance*, sec = second, S = senor (S1 = sensor No.1, S2 = sensor No.2,,,,,,, S5 = sensor No.5).

The change in the electrical resistance in each sensor was analyzed according to Fig. 1**.**

(I) The point at which the resistance was lowest during each heat release from the voltage
(II) heater (min: min1, min2, ……, min5) of every sensor.
(III) The difference of the electrical resistance before and after the electric discharge at each time (gap: gap1, gap2, ……, gap5) of every sensor.
(IV) The period which the electric resistance before and after the electric release in each time (t: t1, t2, …., t5) of every sensor.
(V) The slope of the time and the decrease in the electrical resistance (the highest to the lowest point) plotted against each other at each time (sl: sl1, sl2, …., sl5) in every sensor.

### Clinical allocation and assessment

The authors classified the sample population into two groups, which were the cancer group and the non-cancer group to identify the potential change in the electrical resistance of each sensor of the patients with genitourinary cancer. The cancer group patients consisted of renal cell carcinoma, transitional cell carcinoma (bladder cancer), and prostatic cancer (adenocarcinoma). The non-cancer group consisted of normal subjects and patients with other renal diseases such as primary glomerular diseases and lupus nephritis. The collective data was obtained among the patients and normal subjects with the age > 15 years and estimated glomerular filtration rate > 60 mL/min/1.73 m^2^. For the non-cancer group, all the participants would be examined and confirmed with pathology, and they did not previously have any other cancers in other systems.

The comparison of the electrical resistance of each sensor between the cancer group and the non-cancer group was done using 4 parameters which were “min”, “gap”, “time”, and “slope”. Those parameters were recorded during the changes of the electrical resistance during the time that metal oxide sensors detecting the target VOCs in five different periods that the changes of temperature occurred from the voltage heater. Therefore, all 4 parameter variables from each change of the temperature from the voltage heater in one individual sensor added up to be 20 variables which were (min1, gap1, t1, sl1, min2, gap2,.., min5, gap5, t5, SL5). Moreover, there were a total of 100 variables when all 5 sensors were considered in this study.

### Statistical analysis

The researchers employed the R software version 2.9.1 and SPSS 18.0 statistical software package (SPSS Inc., Chicago, IL, USA) in data analyses. After the analyses had been done, continuous variables were presented in the form of mean ± standard deviation (SD), and categorical variables were presented in the form of percentages. The differences between the two groups were compared by use of the Student’s t test and the Pearson chi-square test for the continuous and categorical variables, respectively. The Spearman correlation coefficient was used for assessing the correlation of selection algorithms of continuous and ordinal variables. The area under the Receiver Operating Characteristic curve (AUC ROC) was used in measuring diagnostic accuracy and p-values ≤ 0.05 were considered statistically significant.

### Ethics declaration

The clinical and demographic data was collected retrospectively. Informed consent was obtained from all individuals for the use of their clinical data. The protocol for the patient's participation was approved by the Human Research Ethics Board of Suranaree University of Technology (Issue # EC-64–109) and performed by relevant regulations and guidelines.

### Institutional review board statement

The participation protocol was approved by the Human Research Ethics Committee, Suranaree University of Technology (Issue # EC-64-109).

### Informed consent statement

Informed consent was obtained from all subjects involved in the study.

## Results

### Participants’ characteristics

The authors assessed urinary volatile organic compounds (VOCs) in a cohort of 64 participants between December 2021 and September 2023 at Suranaree University of Technology Hospital, Thailand (Table 1). The participants were divided into two groups: a cancer group and a non-cancer group. The genitourinary cancer group included 32 patients, consisting of renal cell carcinoma (3.1%), transitional cell carcinoma of the bladder (46.9%), and prostate cancer (adenocarcinoma) (50%). The non-cancer group also had 32 patients, with 9 being healthy subjects and 23 having other genitourinary diseases, such as lupus nephritis (2 patients), primary glomerular diseases, including membranous nephropathy, IgA nephropathy, minimal change disease (5 patients), and benign prostatic hypertrophy (16 patients).

**Table 1:** Clinical characteristics of participants.

Clinical characteristic	Cancer group (N = 32)	Non-cancer group (N = 32)	*P* values
Age (year, mean ± SD)	70 ± 10 (60–80)	58 ± 14 (44–72)	< 0.001
Gender (% male)	42.2%	32.8%	0.83
BMI (kg/m^2^, mean ± SD)	28 ± 4	26 ± 3	0.08
eGFR by CKD-EPI (ml/min, mean ± SD)	82 ± 20 (62–102)	93 ± 23 (70–116)	0.05
Urine specificity (mean ± SD)	1.014 ± 0.006 (1.008–1.020)	1.013 ± 0.007 (1.006–1.020)	0.56

Among the participants, the mean age at the time of evaluation was 70 years, with 42.2% being male. The estimated glomerular filtration rate (eGFR) of the study population was 82 mL/min. The researchers specified that all participants must have an eGFR greater than 60 mL/min to prevent the potential influence of impaired GFR due to advanced chronic kidney disease or acute kidney injury on the analysis of urine VOCs.

Participants who were not normal subjects required confirmation of their diagnosis through patho-histology to be included in the cancer group. Within the cancer group, 35 patients were in stages 1–2, while patients with primary glomerular diseases and lupus nephritis received standard medications, including corticosteroids, mycophenolate mofetil, and calcineurin inhibitors (CNIs; cyclosporine or tacrolimus), along with cyclophosphamide. In the cancer group, patients who were selected were those who had not yet responded to treatment and were undergoing chemotherapy, including Cisplatin and Gemcitabine. Importantly, patients in the cancer group had to have no comorbidities that present in the non-cancer group and had to have cancer in the same organ only, ensuring precise diagnostic interpretation.

When comparing the general characteristics of both groups, as shown in Table 1, it was observed that the patients in the cancer group were significantly older than those in the non-cancer group (*P* < 0.001). However, no other statistically significant clinical characteristics were identified between the study groups.

### Potential Urine VOCs determine genitourinary cancer

In the study's objectives, which aimed to find comparative data for screening urine VOCs in patients with genitourinary cancers (KUB) and compare them with VOCs in a normal population that may also have other non-cancer-related diseases, the researchers examined to identify significant variations in electrical resistance changes within semiconductors, following the methodology protocol, among three distinct groups: cancer patients, non-cancer patients (with other renal diseases), and normal subjects.

The findings revealed significant differences in the alteration of electrical resistance, particularly in sensors S2 and S4, for specific parameters. However, no significant differences were observed between the population groups in S1, S3, and S5 across all parameters (details are provided in the Supplementary data). It was observed that the electrical resistance of VOC samples from patients differed from those of non-cancer patients and normal subjects in sensors 2 (methane, iso-butane, hydrogen, ethanol). This variation was evident when applying a voltage heater at 2500 mV and measuring parameters called "min" (the lowest point of the electrical resistance), "gap" (the delta resistance changes), "t" (time from the highest to the lowest point of the electrical resistance), and "SL" (the slope of the graph plotting time against the decrease in electrical resistance). Furthermore, at 3500 mV in sensor 2, differences were identified in the parameters "gap," "t," and "SL," while at 4500 mV, differences were observed in all three study groups, particularly in the "SL" parameter.

Additionally, for sensor 4 (Ethanol, Hydrogen, Methane, Isobutane, Propane) at the 2000 mV level, the "min" parameter was significant. For sensors 1, 3, 5, and other parameters of S2 and 4, no significant differences were found among the study groups.

### Receiver operating characteristic (ROC) analysis for predicting KUB cancers from others (non-cancer disease and normal subjects)

To achieve the study's objectives, which involved utilizing urine VOCs as a screening biomarker tool to distinguish KUB cancers from non-cancer diseases and normal subjects, the researchers calculated the area of ROC analysis for sensor 2 in all parameters. This choice was made based on the results of the T-test, which indicated significant differences in sensor 2 across several parameters (gap, t, SL). In contrast, for the other sensors, only sensor 4 parameter "min" at the 2000 mV voltage heater level was considered. To facilitate the analysis, the non-cancer disease and normal subject groups were combined into a single "non-cancer group," enabling binary data to be used in ROC analysis (Table 2).

**Table 2:** Receiver operating characteristic (ROC) analysis

Variable(s)	AUC	95% Confidence interval		***P*** value
		Lower bound	Upper bound	
min1S4	0.64	0.50	0.77	0.06
**gap1s2**	**0.64**	**0.50**	**0.78**	**0.04**
gap2s2	0.58	0.44	0.73	0.25
gap3s2	0.60	0.46	0.74	0.16
**gap4s2**	**0.64**	**0.50**	**0.78**	**0.04**
gap5s2	0.62	0.48	0.76	0.10
gap6s2	0.56	0.41	0.70	0.44
T1S2	0.50	0.36	0.64	1.00
T2S2	0.44	0.29	0.58	0.38
T3S2	0.43	0.29	0.57	0.33
T4S2	0.44	0.30	0.59	0.43
T5S2	0.47	0.32	0.61	0.66
T6S2	0.47	0.35	0.61	0.67
SL1S2	**0.64**	**0.50**	**0.78**	**0.04**

ROC = Receiver Operating Characteristic, AUC = the areas under the ROC curve

The AUC values were instrumental for predicting the differentiation between cancer and non-cancer cases by utilizing a semiconductor-based approach for detecting potential Urine VOCs to determine Genitourinary Cancer.

Significant values are in bold.

It was found that sensor 2, which detected VOCs in the methane, iso-butane, hydrogen, and ethanol groups, at a voltage heater of 2000 mV with parameters gap (the delta resistance changes) and Sl (the slope of the graph plotting time against the decrease of electrical resistance) showed a significant area under the ROC curve (AUC) that could be used to distinguish cancer from non-cancer. In contrast, there was no significant AUC for sensor 4 at parameter min (the lowest point of electrical resistance) when using a voltage heater of 2000 mV to differentiate cancer from non-cancer. This observation was made through ROC analysis when diagnosing cancer compared to the non-disease group, as previously observed in the t-test analysis.

### The cut point of electrical resistance of VOCs to differentiate KUB cancer

For determining the Cut Point of Electrical Resistance of VOCs to distinguish KUB cancer, further analysis was conducted using ROC to find the cut-off point based on the Youden's index, which captured the performance of a dichotomous diagnostic test. From the VOC parameters with significant AUC in distinguishing cancer from non-cancer, the sensitivity, specificity, positive predictive value (PPV), negative predictive value (NPV), and accuracy of each parameter were determined. These cut-off points from the Youden's index were selected from the potential parameters that demonstrated the best performance in the diagnostic test, providing the highest accuracy for each parameter, as presented in Table 3.

**Table 3:** Receiver operating characteristic (ROC) analysis

Variable(s)	Cut off ≥	Sensitivity (%)	Specificity (%)	PPV (%)	NPV (%)	Accuracy (%)
gap1S2 ≥	*30	40.6	87.5	76.5	59.6	**64.1**
gap4S2 ≥	*862	75.0	59.4	64.9	70.4	**67.2**
SL1S2 ≥	*0.074	40.6	87.5	76.5	59.6	**64.1**

Youden’s index Sensitivity Specificity positive predictive value (PPV) negative predictive value (NPV) and Accuracy the potential Urine VOCs determine Genitourinary Cancer.

Significant values are in bold.

### The target urine VOCs for screening genito-urinary cancer

**Figure 2:**
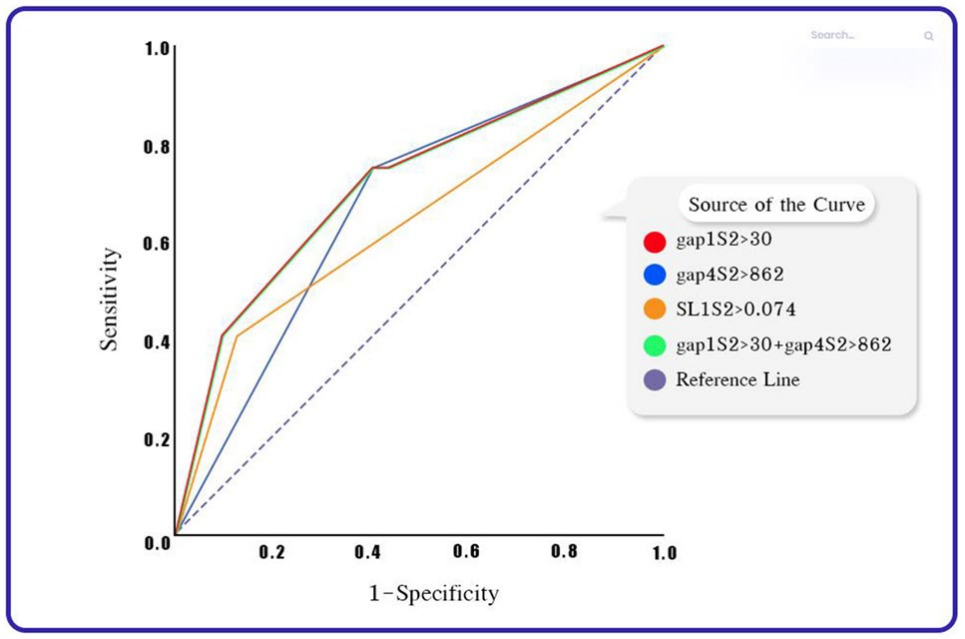
Comparing the areas under the ROC curve (AUC); It is found that using a combination of parameters, which were "gap1S2" with a cut point ≥ 30 and "gap4S2" with a cut point ≥ 862, yields a higher AUC (0.72) compared to using VOC parameters alone in predicting the occurrence of genitourinary cancer. However, the three parameters, including "gap1S2," "gap4S2," and the combined parameter "gap1S2 + gap4S2," showed statistical significance in predicting the occurrence of the disease, with p < 0.05.

The researchers utilized the cut-off points obtained from Youden's index, as presented in Table 3, to perform logistic regression analysis. The analysis revealed that all three potential VOC parameters displayed statistically significant correlations when predicting genito-urinary cancer in patients. Notably, the parameters gap1S2 and SL1S2 exhibited collinearity, meaning they had similar characteristics, as evidenced by the consistent results from logistic regression and ROC analysis. Consequently, the researchers chose to incorporate gap1S2 with a cut-off point of ≥ 30 and gap4S2 with a cut-off point of ≥ 862 to create a new biomarker for analysis. These new biomarkers were used to find a significant association when predicting genito-urinary cancer in patients, and its area under the ROC curve (AUC) was greater compared to using individual VOC parameters for the diagnosis of genito-urinary cancer, as shown in Table 4 and Fig. 2. When comparing the use of the combined parameters gap1S2 and gap4S2 with the individual parameters gap1S2 and gap4S2, the AUC was found to be 0.72, 0.64, and 0.67, respectively. All three of these parameters were statistically significant in their predictive capabilities (*P* < 0.05).

**Table 4:** Logistic regression and ROC analysis of potential urine VOCs for diagnosis of the genito-urinary cancer.

Potential Urine VOCs Parameters for Detecting Genito-Urinary Cancer	OR	Multivariate Analysis (Logistic Regression Analysis)			ROC Analysis			
		95% Confidence Interval		*p-value*	**Area Under ROC**	95% Confidence Interval		p-value
		Lower Bound	Upper Bound			Lower Bound	Upper Bound	
gapS2 ≥ 30	4.79	1.35	16.94	0.015	0.64	0.50	0.78	0.04
gap4S2 ≥ 862	4.39	1.51	12.74	0.007	0.67	0.54	0.81	0.02
SL1S2 ≥ 0.074	4.79	1.35	16.94	0.015	0.64	0.50	0.78	0.04
**gap1s2 ≥ 30 + gap4s2 ≥ 862**	**3.86**	**1.33**	**11.16**	**0.013**	**0.72**	**0.59**	**0.84**	**0.003**

VOCs = Volatile Organic Compounds, ROC = receiver operating characteristic curve, OR = Odd ratio.

Significant values are in bold.

## Discussion

The previous reports discussed the use of urine VOCs for detecting various cancers such as breast cancer, bladder cancer, and prostatic cancer. The research primarily focused on identifying target VOCs specific to each disease and employed gas chromatography as the main technique for detecting these biomarkers. However, the specialized measurement process mentioned previously might impact the sensitivity of screening and could be too costly to be used for screening. The study aimed to develop VOC detection using commercially available semiconductors to make cancer screening in the genitourinary tract more efficient. This measurement approach involved a panel detection method, as multiple target VOCs can change in different cancer types. The results appeared to resemble an "odor imprint" of the VOC panel specific to each disease, as the interaction between metal oxide and semiconductor may vary.

The study aimed to study the various types of cancer by using non-invasive screening that also combined VOC panels specific to each disease. These findings indicated that VOC measurement had enhanced sensitivity and was suitable for screening to detect cancer compared to the approach of individually testing target VOCs, as commonly done in gas chromatography. Furthermore, temperature variations had a significant impact on the release of electrons from metal oxides when they interacted with target VOCs. This, in turn, led to changes in the electrical current within the semiconductor. Consequently, the researchers conducted a study where they established a protocol for altering the temperature within the semiconductor channel using voltage heaters, with varying durations and voltage levels. They then measured the changes in electrical resistance by analyzing the slope of the graph (as shown in Fig. 1) and the delta change in electrical resistance before and after applying the electric current. The alteration in these parameters proved to be specific to different target VOCs associated with various diseases, thereby enhancing the specificity of semiconductors in disease diagnosis.

### Semiconductor for screening genito-urinary cancer

Previous research had found correlations between Renal Cell Carcinoma (RCC) and the detection of urine VOCs in the phenol group (Benzenol), aldehydes such as decanal and nonanal, benzene amine or benzene compounds like aniline and styrene, as well as organosilandiols, which were the silicon analogs to alcohols, including dimethylsilanediol^8^. In contrast, transitional cell carcinoma had shown associations with alkanes, aldehydes, and aromatic compounds like 1-Methylnaphthalene, 2-Methylnaphthalene, and 1,2,4-Trimethylbenzene^9^. For Prostatic Carcinoma, a relationship had been established with pentadecane, alkane hydrocarbon, decanoic acid, and carboxylic acid^20^. It was found that all three cancer types share common features involving hydrogen and ethanol structures, which could be detected by sensors designed for hydrogen and ethanol target gases, as utilized in sensors 2 and 4. This data supported the idea that semiconductor devices employed in screening for genitourinary cancer should focus on target gases within the Ethanol and Hydrogen categories.

### Electrical pattern for detecting genitourinary cancer

The changes in electrical resistance, which were defined as a parameter for distinguishing genitourinary cancer from other urinary tract conditions, were significant. Furthermore, the distinct types of metal oxide had an impact on these electrical resistance parameters. This study revealed that differences in electrical resistance before and after temperature changes using a voltage heater or gap (referred to as the delta resistance from the maximum before temperature change to the minimum after temperature change) provided specific characteristics for identifying genitourinary cancer compared to other conditions. Even though various diseases might have yielded VOCs with overlapping spectrums due to the varying concentrations of these compounds in different diseases, the specific distinctions between the 2000 and 4500 mV voltage currents indicated that the VOC characteristics in genitourinary cancer were unique in their interaction with metal oxides compared to other diseases.

Additionally, this research introduced commercial semiconductor devices that were readily available in the market, which were economically advantageous when compared to gas chromatography detection methods using metal oxides like Tin (Sn). Shifting to different metal oxides might have resulted in varying levels of sensitivity and specificity.

In summary, the analysis of various types of cancer and the combination of screening and comprehensive analysis, as in this study, could result in a differing result. Nevertheless, it was possible to affirm that cost-effective semiconductor devices, which were cheaper than gas chromatography, could play a role in cancer screening. However, the author did not have any intention to replace the use of standard tissue diagnosis in screening the cancer. The benefit of the VOC panels could overcome the biomarker methods which varied according to the cancer cell type.

### Limitation of study and further research recommendation

While there were comparative study reports on both sensitivity and specificity, including the validation of metal oxide sensors and gas chromatography, which was commonly used as the gold standard and deemed to be efficient, especially in terms of sensitivity, leading to its application in disease screening or medical substance detection^15,16^ were limited. Additionally, the specimen preparation processes for both techniques differed, making it challenging to assess cost-effectiveness, particularly concerning temperature control. The need for designing tools with a closed system to prevent external temperature or air interference added to the difficulty. The researchers hoped that there would be more reports in the future regarding the use of metal oxide sensors in medical diagnosis, especially in terms of cost-effectiveness. Nevertheless, the advantages of measuring VOCs in the form of electrical resistance changes with metal oxide sensors, providing numerical results, might have facilitated easier development into AI compared to the analysis in the chemical shift format used in gas chromatography's Flame Photometric Detector system. This suggested that analyzing with metal oxide sensors might have been advantageous for advancing AI development in the future.

### Supplementary Information

Supplementary Information.

## Data Availability

The data that support the findings of this study are available from the corresponding author, CK., upon reasonable request.

## Abbreviation

VOCs: Volatile organic compounds
